# Prefrontal Glutathione Levels in Major Depressive Disorder Are Linked to a Lack of Positive Affect

**DOI:** 10.3390/brainsci13101475

**Published:** 2023-10-19

**Authors:** Ruth O’Gorman Tuura, Andreas Buchmann, Christopher Ritter, Adrian Hase, Melanie Haynes, Ralph Noeske, Gregor Hasler

**Affiliations:** 1 Center for MR Research, University Children’s Hospital, 8032 Zürich, Switzerland; 2 Psychiatry Research Unit, University of Fribourg, 1752 Villars-sur-Glâne, Switzerland; 3 Translational Research Center, University Hospital of Psychiatry and Psychotherapy Bern, 3012 Bern, Switzerland; 4 GE HealthCare, 80807 Munich, Germany

**Keywords:** depression, oxidative stress, glutathione, magnetic resonance spectroscopy, mood, biomarker

## Abstract

Major depressive disorder (MDD) is one of the most common neuropsychiatric disorders, with symptoms including persistent sadness and loss of interest. MDD is associated with neurochemical alterations in GABA, glutamate, and glutamine levels but, to date, few studies have examined changes in glutathione (GSH) in MDD. This study investigated changes in GSH in an unmedicated group of young adults, including 46 participants with current (*n* = 12) or past MDD (*n* = 34) and 20 healthy controls. Glutathione levels were assessed from GSH-edited magnetic resonance (MR) spectra, acquired from a voxel in the left prefrontal cortex, and depressive symptoms were evaluated with validated questionnaires and clinical assessments. Cortisol levels were also assessed as a marker for acute stress. Participants with current MDD demonstrated elevated GSH in comparison to participants with past MDD and controls, although the results could be influenced by differences in tissue composition within the MRS voxel. In addition, participants with both current and past MDD showed elevated cortisol levels in comparison to controls. No significant association was observed between GSH and cortisol levels, but elevated GSH levels were associated with a decrease in positive affect. These results demonstrate for the first time that elevated GSH in current but not past depression may reflect a state rather than a trait neurobiological change, related to a loss of positive affect.

## 1. Introduction

Major depressive disorder (MDD) is one of the most common neuropsychiatric disorders worldwide, with an estimated lifetime prevalence of 10.8% [1], increasing to 19% among adolescents [2]. Symptoms of MDD include a persistent sadness and low mood, anhedonia (loss of pleasure, loss of interest), irritability, low energy levels, altered appetite, and disrupted sleep [3]. However, MDD is clinically heterogeneous, with symptoms varying between participants or within a participant over time, possibly due to differences in stress reactivity [4] or inflammation [5]. Understanding the neurochemical changes associated with depression and the role of stress and inflammation in MDD is important for developing targeted and effective therapies and improving treatment response.

Magnetic resonance spectroscopy (MRS) studies of depression have revealed neurochemical alterations in GABA, glutamate, glutamine, and choline levels [6,7]. However, to date, few studies have examined changes in glutathione in MDD. Glutathione is a tripeptide synthesized from glutamate, cysteine, and glycine and acts as an antioxidant and plays an important role in cell signaling, differentiation, and proliferation and gene expression and protein function [8]. It is also a marker for oxidative stress, which has been suggested to play an important role in the pathogenesis of MDD [9]. Glutathione exists in both oxidized (GSSG) and reduced forms (GSH), but glutathione levels in the human brain appear to consist predominantly of reduced glutathione (GSH) [10].

In the context of depression, GSH is of particular interest since accumulating evidence suggests that depression is associated with chronic low-grade inflammation leading to increased oxidative stress [11]. Antidepressant medications like selective serotonin reuptake inhibitors (SSRIs) can decrease the production of pro-inflammatory cytokines like tumor necrosis factor (TNF)α and interleukin (IL)-1 and increase the production of anti-inflammatory cytokines such as IL-10 and transforming growth factor β1 (TGFβ1) [12]. Remission of depression is associated with a normalization of these and other inflammatory markers [13], while chronic depression is associated with a resistance to these antidepressant effects, possibly as a result of chronic inflammation or the effects of chronic oxidative stress. It is shown in [14,15] that GSH levels are sensitive to inflammation via multiple mechanisms. GSH synthesis is regulated by the availability of glutamate cysteine ligase (GCL) [16], which in turn is regulated by TGFβ1, which decreases GCL expression, such that high TGFβ1 levels lead to reduced GSH production [16]. TGFβ1 levels are reduced in MDD, and TGFβ1 has been suggested as a pharmacological target for major depression [17]. However, in addition to affecting GSH production, inflammation also affects GSH availability, as GSH acts as a scavenger for free radicals and neutralizes reactive oxygen species generated during an inflammatory response [18]. GSH levels, therefore, depend both on the inflammatory response and the degree of oxidative stress, but the interplay between these two processes is complex [19] and has not yet been fully elucidated [18]. 

Measurement of GSH in vivo with MRS is challenging due to the low concentration of GSH and the overlapping resonances from other neuro-metabolites, but recent advances have demonstrated that GSH can be measured reliably with spectral editing methods, even at standard clinical magnetic field strengths [10,20,21]. However, MRS studies investigating GSH changes in depression have shown mixed results. Two previous MRS studies reported decreased occipital GSH levels in unmedicated patients with MDD [22,23], and another observed an inverse association between occipital GSH and anhedonia severity, such that lower GSH levels were associated with higher anhedonia scores [24]. In the prefrontal cortex, one post-mortem study reported significantly decreased GSH in MDD [25]. A recent open-label study demonstrated that transcranial magnetic stimulation increased GSH levels in the left dorsolateral prefrontal cortex in 18 unmedicated patients with MDD, although there was no correlation between the GSH increase and the decrease in depression severity after treatment [26]. However, in contrast to the decrease in occipital and frontal GSH levels reported in MDD in other studies, Duffy et al. [27] reported increased anterior cingulate GSH levels in older adults at risk for depression and a positive association between GSH levels and depressive symptoms [27]. Increased frontal GSH levels have also been reported in the context of bipolar disorder [28] and PTSD [29] (for a recent review, see: [8]) and have been related to increased sleepiness or gait problems post-concussion [30,31], possibly reflecting an increased inflammatory response [30]. However, to date, no previous studies have examined GSH changes in a sample of remitted as well as depressed participants in comparison to controls, and it is not known whether changes in GSH state change rather than a trait change or how GSH levels relate to stress markers like cortisol. The aim of this study was to investigate changes in frontal GSH in a largely medication-naïve group of participants with current and past depression and healthy controls and to investigate the link between GSH, stress markers, depressive symptoms, and mood.

## 2. Materials and Methods

Glutathione-edited spectra were acquired from 66 young adults (mean age = 25 years, range = 18–39) including 20 healthy controls, 34 participants with past MDD, and 12 participants meeting the diagnostic criteria for current MDD. Participants were recruited as part of a larger study investigating GABAergic and glutamatergic changes in depression using MRS [7]. Both healthy participants and those with current or past MDD were recruited from the community via advertisements in local newspapers or university blackboard pages. Study procedures included a telephone pre-screening to check for eligibility according to the inclusion/exclusion criteria, a battery of online psychological and demographic questionnaires, a face-to-face assessment including a structured clinical interview and further clinical assessments, and a magnetic resonance imaging (MRI)/MRS session (see subsections below for details). Blood and saliva samples were also taken prior to the MRI scan. All participants gave verbal and written informed consent to participate in the study, which was conducted under ethical approval from the cantonal ethics committee of the canton of Zürich, Switzerland (KEK-ZH-2012-0381, BASEC: PB_2016-01595).

Participants were eligible for inclusion in the study if they were aged 18–39 years without contraindications for MRI. Exclusion criteria included a history of neurological or severe physical disorders (e.g., heart disease) or a positive history or family history of severe psychiatric disorders, such as psychosis, substance use disorder, and acute eating disorders. A total of 62 of the 66 participants were medication-naïve for psychotropic medication prior to the study. One participant with current MDD had previously taken antidepressants and two participants (one with current MDD and one with past MDD) were prescribed methylphenidate (*n* = 1) or dexamphetamine (*n* = 1) for comorbid ADHD. In addition, one participant with past depression had been prescribed a sedative to use to aid sleep after shift work (once every two weeks). All participants on psychotropic medications withdrew from medication for a minimum of 3 months prior to the measurements. A total of 14 participants were taking contraceptives and 18 regularly took non-prescription anti-inflammatory or analgesic medications (e.g., ibuprofen, paracetamol). Other medications included anti-allergy medications (*n* = 5, taken as needed; 2 control, 1 past MDD, and 2 current MDD), anti-reflux medication (*n* = 1 past MDD), an antiretroviral for HIV prevention (*n* = 1 past MDD), prostaglandin analog eye drops (*n* = 1 past MDD, for retinal thinning), and a diuretic (*n* = 1 past MDD, for Gitelman syndrome).

### 2.1. Data Acquisition and Analysis

**Figure 1:**
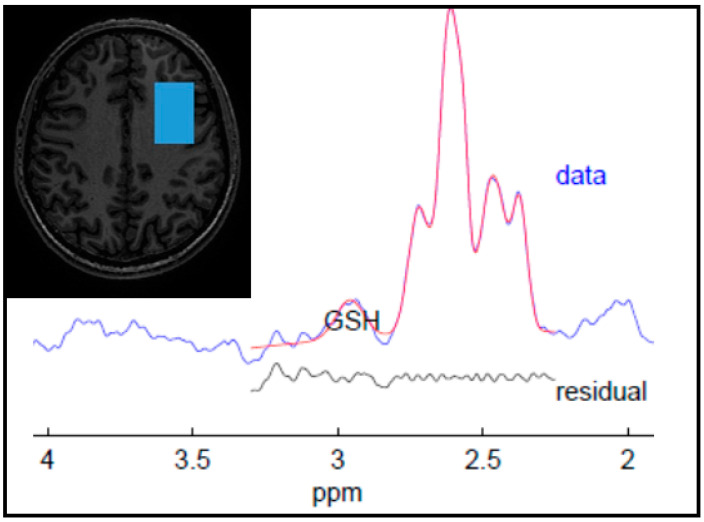
Screenshot showing the position of the left prefrontal voxel (in radiological orientation), and a representative spectrum, analyzed in Gannet [35]. The spectral data are shown in blue with the fit overlaid in red. The residual is displayed beneath the spectrum.

MRI and MRS data were collected with a GE 3T Discovery MR750 MRI scanner, using an 8-channel head coil. GSH-edited spectra were collected from a 25 × 40 × 30 mm^3^ voxel centered in the left dorsolateral prefrontal cortex (see Figure 1 for voxel position), with the MEGAPRESS method (echo time (TE) = 131 ms, repetition time (TR) = 1800 ms, 128 edit ON/OFF pairs). The dorsolateral prefrontal cortex was selected since it appears to be a key neural substrate for depression [32], and the left side was selected due to the default chemical shift direction on the scanner used for MRS data collection, whereby fat is shifted to the left and water to the right, so that both scalp fat and ventricular water are shifted away from the voxel. The center of the spectrum was localized in the dorsolateral prefrontal cortex by a standardized set of measurements, as described previously [7,33,34]. Glutathione was edited selectively with editing pulses applied at 4.56 and 20 ppm. A 3D inversion recovery (IR)-prepared spoiled gradient-echo volume was also collected (TE = 5 ms, TR = 11 ms, inversion time (TI) = 600 ms, flip angle = 8 degrees, resolution = 1 × 1 × 1 mm^3^) for planning the voxel position and for subsequent correction for CSF within the voxel [7]. Spectra were pre-processed and analyzed with Gannet version 3.0 [35], using the default SpecRegHermes alignment with 3 Hz line broadening. GSH levels were calculated as water-scaled concentrations. 

### 2.2. Clinical Assessments

The clinical assessments were conducted by a trained psychologist and included the Structured Clinical Interview for DSM-IV Axis I Disorders [36] for establishing MDD diagnoses and the Hamilton Depression Rating Scale (HAM-D; [37]) and the Montgomery–Asberg Depression Rating Scale (MADRS; [38]) for recording the severity of depressive symptoms. Depressive symptom severity was also measured with the self-reported Beck Depression Inventory (BDI; [39,40]). Participants with current depression according to the structured clinical interview were assigned to the “current MDD” group irrespective of whether they also had a history of a previous depressive episode, while all participants in the “past MDD” group were remitted and did not meet the diagnostic criteria for current depression. In addition, trait positive and negative affect (mood) were assessed with the Positive and Negative Affect Schedule (PANAS; [41]), and perceived stress was assessed with the Perceived Stress Scale (PSS; [42]). As an additional marker for acute stress, serum cortisol levels were derived from venous blood samples taken by a trained medical-technical assistant approximately 20 min before the MR session. Blood samples were centrifuged approximately 60 minutes after collection, and serum cortisol levels were analyzed in an accredited diagnostic laboratory on a Cobas e411 analyzer using a competitive electrochemiluminescence immunoassay (Roche Diagnostics, Rotkreuz, Switzerland) with a coefficient of variation of 3.6% at 793 nmol/L. 

### 2.3. Statistical Analyses

Groupwise differences in GSH, cortisol, symptom scores, and potential confounding variables (age, sex, IQ, and voxel composition) were assessed between the control, past MDD, and current MDD groups with a Kruskal–Wallis test using Dunn tests as post hoc pairwise comparisons. The links between GSH levels and symptom scales were assessed with Spearman correlations. Statistical analyses were performed with SPSS 27.0, with a significance level of *p* < 0.05. The main Kruskal–Wallis tests were visualized using the ggbetweenstats() function of the ggstatsplot package [43] in R, version 4.1.2.

## 3. Results

Group demographics are shown in Table 1, together with summary statistics for the GSH and cortisol levels in each group (current MDD, past MDD, and control) and summary statistics for the symptom severity scales. Participants with current and past MDD were significantly younger than controls, but the proportion of males and females was comparable across the three groups. Participants with current depression showed significantly higher scores on all scales of depression severity, higher levels of perceived stress, a higher proportion of comorbid anxiety disorders, and a more negative mood profile (Table 1). Participants were highly educated on average, with a mean of 13–14 years of education, but education levels did not differ significantly between the groups. There were also no significant groupwise differences in performance on a multiple choice vocabulary intelligence test (MWT-A), as a marker for IQ [44].

**Figure 2:**
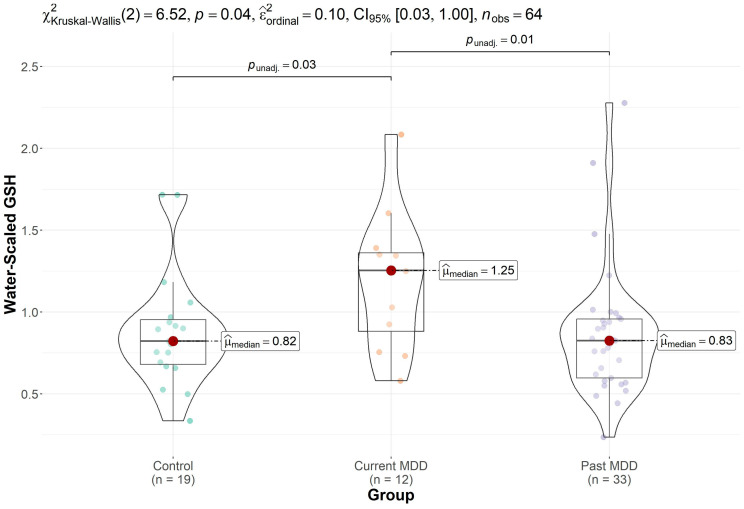
Boxplot depicting the median and interquartile ranges of the water-scaled GSH levels in each group, after removal of two outliers (one control and one participant with past depression). GSH levels were elevated in the participants with current depression in comparison to those with past depression and controls (*p* = 0.038, Kruskal–Wallis test).

Trend-level groupwise differences in GSH were observed (*p* = 0.074), which became significant (*p* = 0.038, Kruskal–Wallis test) after removal of two statistical outliers (one control and one participant with past MDD) with GSH levels more than four times the group median values and more than seven standard deviations away from the group means. Post hoc comparisons showed elevated GSH levels in the participants with current depression (Figure 2), which were significantly increased in comparison to GSH levels in the controls (*p* = 0.034) and participants with past depression (*p* = 0.013). No differences in GSH levels were evident between controls and participants with past MDD (*p* = 0.846). Groupwise differences in GSH remained significant after excluding the participant taking antidepressants (*p* = 0.013) and after excluding an additional three participants taking any psychotropic medication prior to the study (*p* = 0.005). All participants on psychotropic medications were unmedicated for at least 3 months prior to the measurements. 

The grey matter fraction within the MRS voxel was also significantly different between groups (*p* = 0.017), and post hoc tests revealed a significantly higher grey matter fraction in the current MDD group in comparison to both the past MDD group (*p* = 0.033) and the control group (*p* = 0.005). Although the groups differed in age (*p* = 0.010), no significant correlation was observed between GSH levels and age (rho = −0.141, *p* = 0.267). No significant correlation was observed between the grey matter fraction within the voxel and the GSH concentrations, although a positive trend was seen between GSH levels and the grey matter fraction (rho = 0.236, *p* = 0.061). 

**Figure 3:**
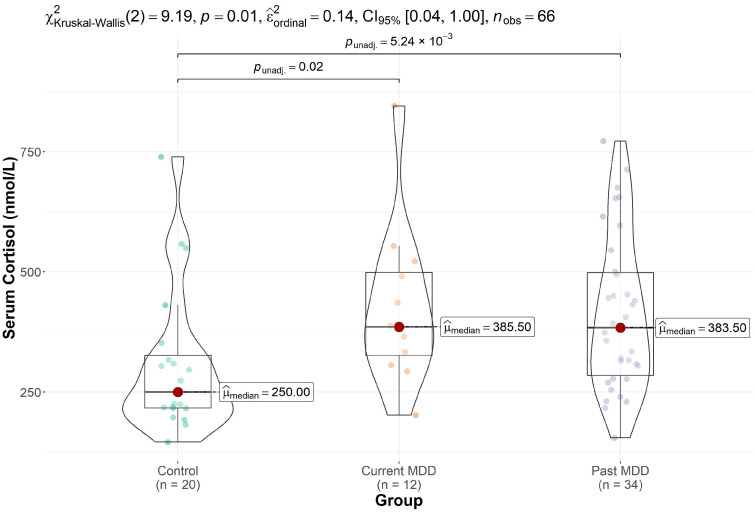
Boxplot depicting the median and interquartile range of the cortisol levels in each group. Cortisol levels were significantly elevated in the participants with current depression and past depression in comparison to controls.

Groupwise differences in cortisol were also significant (*p* = 0.010, Kruskal–Wallis test). Post hoc comparisons revealed elevated cortisol levels in participants with current (*p* = 0.017) and past MDD (*p* = 0.005) relative to controls (Figure 3) but no significant differences in cortisol levels between the two MDD groups (*p* = 0.799). 

**Figure 4:**
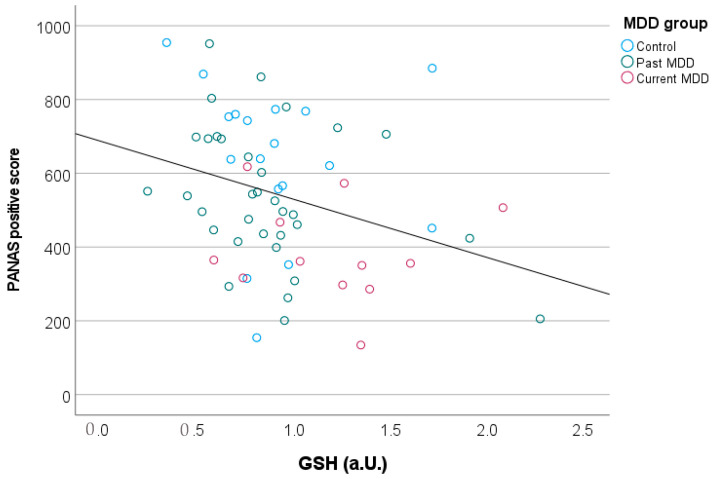
Elevated prefrontal GSH levels were associated with reduced PANAS-positive scores, reflecting a loss of positive mood in participants with higher GSH concentrations.

The correlations with symptom scores revealed a significant negative correlation between GSH levels (across all participants) and a PANAS-positive score (rho (61) = −0.346, *p* = 0.005, Figure 4) and a trend towards a positive correlation with the perceived stress scale (rho = 0.24, *p* = 0.059). The association between GSH levels and positive mood remained significant even after including the grey matter fraction within the voxel as a covariate (rho =−0.310, *p* = 0.014), No significant correlations were observed between GSH and cortisol (rho = −0.086, *p* = 0.497) or between GSH and depression severity (MADRS: rho = 0.144, *p* = 0.261, HAMD: rho = 0.158, *p* = 0.215, BDI: rho = 0.166, *p* = 0.194). 

## 4. Discussion

In this study, we found increased GSH in the prefrontal cortex of unmedicated subjects with current MDD, recruited from the general population. In contrast, GSH levels were not increased in participants with past MDD, suggesting that GSH increases may reflect a state change rather than a trait change.

Previous studies have reported both increases and decreases in glutathione in depression [22,23,24,27]. While decreases in glutathione are typically interpreted as an increase in oxidative stress, the interpretation of increased glutathione is less clear but could represent a compensatory upregulation, potentially protective for increased stress or indicative of stress preconditioning [45]. Alternatively, increases in glutathione could reflect an inflammatory response, as seen following acute concussion [30]. Evidence for such an upregulation has been observed in rodent studies, where mild stress in the form of heat stress, hypoxia, or a mild imbalance between the production and removal of reactive oxygen species has been reported to increase GSH levels (for a recent review, see: [45]). In light of these findings, the elevated prefrontal GSH levels observed in participants with current but not remitted depression may reflect a compensatory upregulation in GSH levels, possibly in response to an increase in perceived stress, although no association between GSH and cortisol levels was observed. Since participants in the present study were high functioning and demonstrated rather mild symptoms, they may have been in a functionally compensated state, in comparison to the patients studied in clinical settings in previous MRS [22,24] and post-mortem [25] studies, where decreased GSH was observed. However, it is also important to consider these findings in the context of the elevated fraction of grey matter within the MRS voxel in the current MDD group. Although post-mortem studies have reported increased levels of GSH in white matter vs. grey matter in humans [46], a recent high-field, multivoxel MRS study reported higher concentrations of GSH in grey matter than in white matter in healthy participants [21]. In our data, we also saw a trend towards a significant positive correlation between the GSH levels and the grey matter fraction within the voxel. We, therefore, cannot exclude the possibility that the elevated GSH observed in the current MDD group may arise partly as a result of the higher grey matter concentration within the MRS voxel. These results should, therefore, be considered with caution until they can be replicated in a larger sample with a more balanced distribution of grey matter fraction between groups.

In addition to an increase in GSH in the current MDD group, we also observed an association between higher GSH levels and a decrease in positive mood, which remained significant after covarying for the fraction of grey matter within the voxel. A putative link between GSH and mood has also emerged in a previous study reporting that chronic treatment with mood stabilizers increases cortical glutathione levels, while treatment with antidepressants does not affect GSH levels [46]. Elevated glutathione levels have also been reported in bipolar disorder [28] and following alcohol consumption [47] where a link between affective behavior and oxidative stress was also observed. 

Unlike in studies of aging, we did not observe a significant association between GSH and age. Previous studies investigating GSH within CSF have reported a pronounced decrease with age [48]. Similarly, a cross-sectional MRS study investigating GSH with edited MRS reported significantly lower GSH levels in an older group of participants (with an average age of 76 years) in comparison to a younger group (with an average age of 20 years) [49]. However, in the present study, the limited age range within the participant group (18–39 years) may have precluded the detection of a significant association with age. 

Other notable differences between the present study and previous studies in the literature include the brain regions under investigation. Most previous studies investigating GSH in depression with MRS have assessed GSH levels in the occipital lobe, reporting decreases in GSH in the context of MDD [22,23]. In contrast, studies investigating the frontal cortex have shown more mixed results, with both increases [27] and decreases [25] reported, possibly due to differences in the methods used to assess GSH, namely in vivo MRS vs post-mortem spectrophotometry. In addition, the age of the participants and the severity of depression differed between studies, such that increases in frontal GSH were reported in older adults with subthreshold MDD symptoms [27], while decreases in frontal GSH were observed post-mortem in middle-aged participants (mean age 46 years) with a history of full-threshold MDD symptoms [25]. Increased frontal GSH might, therefore, be an early compensatory response prior to the onset of more severe symptoms, since increased frontal GSH was reported in participants with subthreshold symptoms in the study by Duffy et al. [27] as well as in our sample of participants with generally mild symptoms. Interest is growing in the therapeutic potential of compounds which could potentially increase GSH levels in stress-related psychopathologies [50], but future studies would be needed to clarify the progression of GSH changes in depression and whether the apparent changes in GSH arise as a cause or consequence of stress or vulnerability to stress-related psychopathology. 

Limitations of the current study include the modest sample size (*n* = 12) for participants with current depression. In addition, the MDD participants typically demonstrated mild symptoms, and cases of severe MDD were underrepresented, which may reduce the effect sizes and the statistical power to detect significant groupwise differences or correlations with symptom scores. Participants were also highly educated, reducing generalizability of the results to the wider population of MDD patients. However, strengths of the study include the community-based recruitment, the unmedicated and mostly medication-naïve MDD group, and the relatively large control sample that matched MDD cases in terms of education and other sociodemographic variables [7]. The inclusion of past MDD allowed for differentiation between state and trait abnormalities in neurobiology related to MDD. In addition, our voxel was placed in the prefrontal cortex in a region associated with the pathogenesis of MDD by other types of evidence.

## 5. Conclusions

Prefrontal GSH concentrations are elevated in participants with current but not remitted depression, possibly reflecting a state change rather than a trait change. In addition, elevated GSH is related to a loss of positive affect, providing further evidence for a link between GSH alterations and anhedonia in affective disorders. Future studies should evaluate GSH in a larger sample including participants with severe as well as mild MDD and explore cerebral GSH levels in the context of inflammatory markers.

## Figures and Tables

**Table 1:** Participant demographics, glutathione (GSH) and cortisol levels, and symptom severity scores stratified by group. Values are given as Mean (SD) or % individuals.

	Control (*n* = 20)	Past MDD (*n* = 34)	Current MDD (*n* = 12)	*p*-Value
Age	27.5 (5.2)	24.7 (4.7)	22.3 (2.8)	0.010
Male sex	37%	30%	42%	0.753
GSH	0.89 (0.36) (n = 19) *	0.87 (0.40) (n = 33) *	1.19 (0.42) *(n = 12)*	0.038
BDI	4.1 (4.3)	8.3 (5.2)	23.1 (9.5)	<0.001
HAM-D (range)	1.83 (4.6) (0–17)	5.42 (4.9) (0–17)	16.3 (9.2) (1–31)	<0.001
MADRS (range)	1.33 (3.8) (0–16)	6.94 (6.1) (0–16)	22.5 (6.6) (13–34)	<0.001
PSS	18.8 (6.1)	25.8 (6.3)	35.7 (5.2)	<0.001
PANAS-positive	638 (211)	540 (185)	386 (135)	0.003
PANAS-negative	53 (80)	83 (102)	222 (171)	<0.001
cortisol	308 (160)	417 (162)	427 (166)	0.013
Voxel GM fraction	0.31 (0.03)	0.32 (0.04)	0.34 (0.03)	0.017
Voxel WM fraction	0.66 (0.04)	0.65 (0.05)	0.63 (0.03)	0.055
Voxel CSF fraction	0.03 (0.01)	0.03 (0.02)	0.03 (0.01)	0.601
Education (years)	13.1 (3.82)	14.0 (3.12)	12.7 (2.16)	0.549
MWT-A	29.2 (4.6)	31.0 (4.6)	29.5 (2.6)	0.280
Comorbid anxiety disorder	21%	55%	75%	0.008

Abbreviations: GSH: glutathione, BDI: Beck Depression Inventory, HAM-D: Hamilton Depression Rating Scale, MADRS: Montgomery–Asberg Depression Rating Scale, PSS: Perceived Stress Scale, PANAS: Positive and Negative Affect Schedule. GM: grey matter, WM: white matter, CSF: cerebrospinal fluid, MWT-A: Mehrfachwahl-Wortschatz-Intelligenztest/Multiple-choice vocabulary intelligence test. Values are given as Mean (SD) or % individuals. * For the GSH levels, two statistical outliers (one control and one participant with past MDD) were excluded. MWT-A values were missing for one participant in the control group.

## Data Availability

Data from the study can be made available upon reasonable request, subject to a data sharing agreement and with appropriate ethical approval.
